# Effects of the sperm DNA fragmentation index on the clinical and neonatal outcomes of intracytoplasmic sperm injection cycles

**DOI:** 10.1186/s13048-020-00658-z

**Published:** 2020-05-02

**Authors:** Linjun Chen, Junshun Fang, Weihua Jiang, Jie Wang, Dong Li

**Affiliations:** Reproductive Medical Center, Nanjing Drum Tower Hospital, The Affiliated Hospital of Nanjing University Medical School, Zhongshan, Road 321#, Nanjing, 210008 People’s Republic of China

**Keywords:** Sperm, DNA fragmentation index, Neonatal outcomes, Intracytoplasmic sperm injection, Birth defects

## Abstract

**Background:**

Most studies have mainly focused on the effects of the sperm DNA fragmentation index (DFI) on fertilization, embryonic developmental potential and aneuploidy, pregnancy and abortion rates after in vitro fertilization (IVF)/intracytoplasmic sperm injection (ICSI) and have remained controversial. However, few studies have reported the effects of sperm DFI on neonatal outcomes, including stillbirths, neonatal deaths, sex, gestational age, prematurity, birthweight, low birth weight (LBW) and birth defects in newborns. Our objective was to evaluate the effects of sperm DFI on the clinical and neonatal outcomes of ICSI cycles.

**Methods:**

This retrospective study analysed a total of 2067 oocyte retrieval, 1139 transfer and 713 delivery cycles from conventional ICSI cycles, including 301, 469, and 214 live-born infants in groups segregated according to sperm DFI as the < 15%, 15–30% and >  30% groups, respectively. The clinical and neonatal outcomes were compared among the three groups.

**Results:**

Sperm DFI did not significantly affect the rates of fertilization, clinical pregnancy, miscarriage or ongoing pregnancy. Sperm DFI did not increase the risk of stillbirths or neonatal deaths. The rates of stillbirths and neonatal deaths were not significantly different among the three groups. The sex, gestational age, prematurity, birthweight and LBW of newborns in the three groups were not significantly affected by sperm DFI. Moreover, sperm DFI did not increase the number of birth defects in children.

**Conclusions:**

Sperm DFI did not affect the clinical or neonatal outcomes of ICSI cycles.

## Background

The integrity of sperm DNA is important for embryonic development and pregnancy. Sperm DNA damage is even frequently found in normozoospermic men [1]. Sakkas et al. [2] reported six main factors (apoptosis, DNA strand breaks, reactive oxygen species (ROS), endogenous caspases and endonucleases, radio- and chemotherapy, and environmental toxicants) that induced sperm DNA damage. The extent of sperm DNA damage is measured by the sperm DNA fragmentation index (DFI).

Santi et al. [3] reported that sperm DFI had a higher accuracy than conventional sperm parameters for sperm functional analysis. Alvarez Sedó et al. [4] reported that sperm DFI was negatively associated with the rates of blastulation and pregnancy in intracytoplasmic sperm injection (ICSI) patients with donated oocytes. Zheng et al. [5] found that sperm DFI had a negative effect on day 3 embryo quality and the rates of blastocyst formation, implantation and pregnancy in patients undergoing in vitro fertilization (IVF). Two meta-analyses showed that high sperm DFI had lower good-quality embryo and clinical pregnancy rates and increased miscarriage rates after IVF/ICSI than did low sperm DFI [6, 7]. However, Sun et al. [8] reported that sperm DFI did not predict embryo quality or pregnancy rate after IVF/ICSI. Sperm DFI was also not associated with blastocyst aneuploidy, morphological grading or clinical outcomes after preimplantation genetic screening [9]. Another two meta-analyses concluded that sperm DFI did not predict IVF/ICSI outcomes [10, 11]. Antonouli et al. [12] reported that sperm DFI did not significantly affect embryonic development or pregnancy rate in ICSI patients with donated oocytes. From the literatures published above, the effect of sperm DFI on embryonic development, implantation and pregnancy remained controversial after IVF/ICSI.

At present, most studies have mainly focused on the effect of sperm DFI on fertilization, embryonic developmental potential and aneuploidy, pregnancy and abortion rates. However, few studies have reported the effect of sperm DFI on neonatal outcomes, including stillbirths, neonatal deaths, sex, gestational age, prematurity, birthweight, low birth weight (LBW) and birth defects in newborns. In addition to the clinical outcomes, our retrospective study compared the effects of different sperm DFIs on neonatal outcomes in ICSI cycles.

## Methods

### Patients

All patients signed informed consent forms for ICSI and follow-up from August 2015 to December 2017 before participating in this retrospective study. All male patients were submitted to the same methods to measure sperm DFI in a sperm chromatin structure assay (SCSA) performed 1–2 months prior to oocyte retrieval. DFI values were used to categorize the subjects into three groups: < 15%, 15–30% and >  30%. Cycles performed in patients with normal chromosomes, who did not receive antioxidant therapy prior to their IVF procedures and underwent day 3 embryo transfer were included in this study. Cycles from day 2 embryo and day 5 blastocyst transfer were excluded from this study. A total of 2067 oocyte retrieval, 1139 transfer and 713 delivery cycles, including 301, 469, and 214 live-born infants from < 15%, 15–30% and >  30% groups, respectively, were studied in this retrospective study.

### Definitions

The published literature [13] was used to define clinical pregnancy, miscarriage, gestational age, prematurity, LBW, live births and stillbirths.

### Follow-up

Data on the clinical outcomes and neonatal outcomes, including clinical pregnancy, miscarriage, the date of birth, sex, live-birth or not, birthweight and birth defects in newborns, were gathered through phone calls and electronic database registration.

### Statistics

SPSS 22.0 software was used for all data analyses. An independent samples Kruskal-Wallis test was used to compare means among the three groups. The χ^2^ test was used to compare rates among the three groups. *P* <  0.05 indicated statistical significance.

## Results

Duration of infertility and male BMI were not significantly different among the three groups. Female BMI in high sperm DFI group (15–30%) was significantly lower compared with that in low sperm DFI group (< 15%), while the distribution of primary infertility and thickness of the endometrium in the 15–30% group were significantly higher compared with that in the DFI <  15% group. Female age and male age were significantly different among the three groups, while sperm DFI significantly increased as female age and male age increased (Table 1), which was consistent with the reported literatures [12, 14].

**Table 1:** Patient characteristics according to sperm DFI

	< 15%	15–30%	> 30%	*p*-Value
Female age (years)	30.9 ± 5.4^a^	30.8 ± 5.3^b^	32.0 ± 5.7^a,b^	< 0.001
Female BMI (kg/m^2^)	22.7 ± 3.1^a^	22.4 ± 3.1^a^	22.7 ± 3.1	0.033
Pattern of infertility				
Primary	408 (59.0)^a,c^	623 (65.4)^a^	260 (61.6)	0.027
Secondary	284 (41.0)	330 (34.6)	162 (38.4)	–
Duration of infertility (years)	4.2 ± 3.3	4.1 ± 3.3	4.6 ± 3.6	0.054
Thickness of endometrium (mm)	10.6 ± 2.9^a^	11.1 ± 3.0^a^	10.8 ± 3.0	0.002
Male age (years)	32.3 ± 5.8^a^	32.7 ± 6.3^d^	34.1 ± 7.4^a,d^	0.001
Male BMI (kg/m^2^)	24.9 ± 3.9	24.8 ± 4.0	24.5 ± 3.9	0.194
DFI (%)	9.8 ± 3.3^b^	22.2 ± 4.3^b^	39.8 ± 9.9^b^	< 0.001

^a,d^*P* < 0.05, ^b^*P* < 0.001, ^c^values in parenthesis are expressed in percentage

The clinical outcomes, including average number of transferred and frozen embryos, the rates of fertilization, biochemical pregnancy, clinical pregnancy, miscarriage and ongoing pregnancy were not significantly different among the three groups (Table 2).

**Table 2:** Clinical outcomes of transfer cycles according to sperm DFI

	< 15%	15–30%	> 30%	*p*-Value
Number of oocyte retrieval cycles	692	953	422	–
Average number of MII oocytes	7.6 ± 4.6^a^	8.2 ± 4.7^a^	8.2 ± 4.9	0.009
Number of fertilized oocytes	4209 (80.3)^b^	6276 (79.9)	2754 (79.9)	0.846
Number of transfer cycles	344 (49.7)^a,c^	558 (58.6)^c^	237 (56.2)^a^	0.002
Average number of transferred embryos	1.9 ± 0.4	1.9 ± 0.3	1.9 ± 0.3	0.621
Average number of frozen embryos	2.33 ± 2.30	2.49 ± 2.36	2.43 ± 2.31	0.276
Number of biochemical pregnancy	247 (71.8)	403 (72.2)	175 (73.8)	0.854
Number of clinical pregnancy	241 (70.1)	386 (69.2)	168 (70.9)	0.884
Number of miscarriage	23 (9.5)	32 (8.3)	20 (11.9)	0.408
Number of termination of pregnancy	2 (0.8)	5 (1.3)	0 (0.0)	0.423
Number of ongoing pregnancy	216 (89.6)	349 (90.4)	148 (88.1)	0.711

^a^*P* < 0.05, ^c^*P* < 0.001, ^b^values in parenthesis are expressed in percentage

There was one stillbirth in each of the DFI <  15% and 15–30% groups, and there was no stillbirth in the DFI >  30% group. There were no significant differences in stillbirths among the three groups. There was one neonatal death in the DFI <  15% group, there were four neonatal deaths in the 15–30% group, and there was one neonatal death in the DFI >  30% group. There was no significant difference in the rate of neonatal deaths among the three groups (Table 3).

**Table 3:** Live births, stillbirths, and neonatal deaths according to sperm DFI

	< 15%	15–30%	> 30%	*p*-Value
Number of delivery cycles	216	349	148	–
Singletons	130 (60.2)^a^	228 (65.3)^b^	82 (55.4)^b^	0.098
Twins	86 (39.8)	121 (34.7)	66 (44.6)	–
Number of live births	301	469	214	–
Singletons	130	228	82	–
Twins	171	241	132	–
Number of neonatal deaths	1 (0.3)	4 (0.9)	1 (0.5)	0.867
Singletons	1 (0.8)	2 (0.9)	0	1.000
Twins	0	2 (0.8)	1 (0.8)	0.611
Number of stillbirths	1 (0.3)	1 (0.2)	0	1.000
Singletons	0	0	0	–
Twins	1 (0.6)	1 (0.4)	0	1.000

^a^values in parenthesis are expressed in percentage

^b^*P* < 0.05

There were no significant differences in the sex or gestational age of newborns among the three groups. Gestational age was significantly larger for twins in the DFI <  15% and >  30% groups than for those in the 15–30% group. There was no significant difference in prematurity among the three groups. The prematurity rate of twins in the DFI <  15% group was significantly lower than that in the 15–30% group. There were also no significant differences in birthweight or LBW in newborns among the three groups (Table 4).

**Table 4:** Neonatal outcomes of live births according to sperm DFI

	< 15%	15–30%	> 30%	*p*-Value
Number of live births	301	469	214	–
Boys	139 (46.2)^a^	219 (46.7)	93 (43.5)	0.731
Girls	162 (53.8)	250 (53.3)	121 (56.5)	–
Gestational age (weeks)	38.0 ± 2.2	38.1 ± 2.4	38.0 ± 2.1	0.461
Singletons	39.0 ± 1.9	39.1 ± 1.6	39.0 ± 1.3	0.282
Twins	36.7 ± 1.9^b^	36.1 ± 2.4^b,c^	36.7 ± 2.2^c^	0.043
Prematurity (< 37 weeks)	78 (25.9)	143 (30.5)	60 (28.0)	0.395
Singletons	10 (7.7)	23 (10.1)	4 (4.9)	0.369
Twins	68 (39.8)^d^	120 (49.8)^d^	56 (42.4)	0.107
Birthweight (grams)	2842.2 ± 661.4	2876.4 ± 665.0	2820.9 ± 584.5	0.411
Singletons	3305.2 ± 562.5	3311.4 ± 534.3	3297.9 ± 468.6	0.826
Twins	2490.3 ± 493.4	2463.1 ± 490.6	2528.3 ± 438.0	0.613
LBW (< 2500 g)	73 (24.3)	115 (24.6)	62 (29.1)	0.386
Singletons	9 (6.9)	10 (4.4)	4 (4.9)^e^	0.575
Twins	64 (37.4)	105 (43.8)^e^	58 (43.9)	0.380

^a^value in parenthesis are expressed in percentage

^b,c,d^*P* < 0.05

^e^an infant with unknown birthweight

For birth defects, there was one birth defect (cleft lip) in the DFI <  15% group, and there were four birth defects (abnormality of the external auditory canal (1), cleft soft palate (1), congenital heart disease (1), and joint deformity of the left index and middle fingers (1)) in the 15–30% group and two birth defects (Down syndrome (1) and an extra finger near the right thumb (1)) in the DFI >  30% group. There were no significant differences in birth defects among the three groups (Table 5).

**Table 5:** Birth defects in live-born children according to sperm DFI

	< 15%	15–30%	> 30%	*p*-Value
Number of birth defects	1 (0.3)^a^	4 (0.9)	2 (0.9)	0.693
	Cleft lip (1)	Abnormality of the external auditory canal (1), Cleft soft palate (1), Congenital heart disease (1), Joint deformity of the left index and middle fingers (1)	Down Syndrome (1), An extra finger near the right thumb (1)	

^a^values in parenthesis are expressed in percentage

## Discussion

This retrospective study showed that sperm DFI did not significantly affect the fertilization, clinical pregnancy, miscarriage, ongoing pregnancy, stillbirths, neonatal deaths, prematurity or LBW rates, gestational age or birthweight of newborns among the different groups.

Sun et al. reported that there were no significant difference in the rates of fertilization, clinical pregnancy or ongoing pregnancy between high (≥ 30%) and low (< 30%) sperm DFI groups from IVF or ICSI cycles [8]. A prospective cohort study showed that sperm DFI was not associated with the fertilization or ongoing pregnancy rate after ICSI cycles [15]. Consistent with the studies mentioned above, our retrospective study found that sperm DFI did not adversely affect the rates of fertilization, clinical pregnancy or ongoing pregnancy in ICSI cycles (Table 2). Zhu et al. reported that the increased sperm DFI was associated with unexplained recurrent pregnancy loss [16]. Yang et al. found that the early abortion rate in intrauterine insemination (IUI) cycles was significantly increased as sperm DFI increased, while there was no significant difference in ICSI cycles [14]. The present study obtained similar results, namely, that the miscarriage rate was not significantly different among different sperm DFI groups (Table 2). This may be related to the fact that spermatozoa showing normal morphology with less DNA damage are used for ICSI [17–19] and that the most viable embryos are chosen for subsequent transfer.

Simon et al. reported that infertile couples with high sperm DFI had a much lower live-birth rate after conventional IVF than infertile couples with a low sperm DFI, while there was no significant difference in live-birth rate in couples undergoing ICSI cycles [20]. A systematic review and meta-analysis also showed that high sperm DFI significantly decreased the live-birth rate from couples undergoing IVF cycles but not from couples undergoing ICSI cycles [21]. This may be related to the following two main reasons [22]. One reason was that female patients undergoing ICSI cycles were younger than those undergoing IVF cycles, and the oocytes had a better ability to repair DNA damage from high DFI spermatozoa. Another reason was that high DFI spermatozoa can produce ROS and expose the oocytes to oxidative assault during sperm-oocyte incubation in IVF cycles, while ICSI did not involve this process. Esteves et al. [23] recommended that live birth must be the most important indicator in the study of sperm DFI. In the present study, we obtained a similar result where high sperm DFI did not increase the risk of stillbirths or neonatal deaths in patients undergoing ICSI cycles (Table 3).

It has been reported that the incidences of prematurity and LBW in newborns are significantly higher after IVF/ICSI than for natural conception [24, 25]. Seggers et al. showed that subfertility-related factors rather than the IVF treatment itself compromised perinatal outcomes [26]. In the present study, high sperm DFI did not significantly increase the risk of prematurity or LBW of newborns from patients undergoing ICSI cycles. Bungum et al. reported that sperm DNA damage was not related to birthweight or gestational age of newborns from patients undergoing IVF/ICSI cycles [22]. Sperm DFI did not significantly affect the sex ratio of newborns. The sex ratio of the high sperm DFI group (> 30%) was slightly lower than that of the other groups. This result may be related to the small sample size of the present study. It may also be related to the fact that Y spermatozoa are more susceptible to stressful conditions than X spermatozoa [27]. High DFI spermatozoa can still fertilize the egg, and subsequent embryo developmental potential and implantation may be impaired, but once a live birth is achieved, neonatal outcomes are not significantly affected.

Sperm DNA damage is often accompanied by numerical and structural chromosomal abnormalities of spermatozoa [28, 29], which can lead to birth defects or congenital malformation in newborns. In the present study, sperm DFI did not significantly affect birth defects of newborns among the three groups. This may be related to the fact that the embryologists would select the spermatozoa with the most normal morphology to inject the oocytes during the ICSI process. This selection was speculated to be related that sperm DFIs from spermatozoa with a normal morphology were significantly lower than those from spermatozoa with an abnormal morphology [17–19]. It was also reported that ICSI performed with spermatozoa selected for better morphology at a high magnification (> 6000×) can achieve better clinical outcomes than conventional ICSI [30, 31]. Some studies found that compared with conventional ICSI, intracytoplasmic morphologically selected sperm injection (IMSI) can decrease the risk of birth defects in newborns [32–34]. On the other hand, this decrease may also be related to the semen processing method. Density gradient centrifugation was used to separate mobile spermatozoa from semen in the present study. Density gradient centrifugation can significantly decrease sperm DFI and the deformity rate [35, 36]. Rouenet al. reported that discontinuous gradient centrifugation can reduce the percentage of unbalanced spermatozoa in semen from chromosomal rearrangement carriers [37]. A live birth with a balanced karyotype was obtained from sperm selection by discontinuous gradient centrifugation for intrauterine insemination in a patient with a chromosomal translocation [38]. Density gradient centrifugation can also remove ROS (including newly generated ROS) and can prevent separated mobile spermatozoa from oxidative stress [39]. Therefore, density gradient centrifugation can achieve spermatozoa with a better morphology and DNA integrity for assisted reproductive treatment. In addition, the fertilized oocytes or zygotes had a certain ability to repair DNA damage [40, 41]. These may be the reasons why high DFI did not increase the risk of birth defects in newborns from patients undergoing ICSI cycles.

In the present retrospective study, ejaculated spermatozoa were utilized for conventional ICSI. To obtain better clinical outcomes, Bradley et al. [42] reported that interventions, such as physiological intracytoplasmic sperm injection (PICSI), IMSI and testicular sperm extraction/aspiration (TESE/TESA), can improve the live-birth rate for patients with high sperm DFI. Among the three interventions, compared with the control spermatozoa, testicular spermatozoa significantly improved the live-birth rate. This may be related to the fact that sperm DFIs from testicular spermatozoa were significantly lower than those of ejaculated spermatozoa [43]. Zhang et al. [44] reported that compared with ejaculated spermatozoa, testicular spermatozoa significantly increased the pregnancy and live-birth rates for patients with high sperm DFIs. Since TESE/TESA is an invasive method, it is generally recommended that testicular spermatozoa are an alternative for patients with high sperm DFI who have failed recurrent ICSI cycles or other less invasive interventions [45]. It has been reported that testicular spermatozoa significantly increase clinical pregnancy and live-birth rates and decrease miscarriages in patients with high sperm DFI who suffer from recurrent ICSI failure [46–48]. Healthy live birth is the most important for patients with high sperm DFI, regardless of testicular spermatozoa or other less invasive interventions.

## Conclusions

Sperm DFI did not compromise the clinical and neonatal outcomes of newborns in our retrospective study. Multicentre and randomized controlled trials are needed to confirm our conclusion.

## Data Availability

Not applicable.

## Abbreviations

DFI: DNA fragmentation index
IVF: In vitro fertilization
ICSI: Intracytoplasmic sperm injection
LBW: Low birth weight
ROS: Reactive oxygen species
BMI: Body mass index
IMSI: Intracytoplasmic morphologically selected sperm injection
PICSI: Physiological intracytoplasmic sperm injection
TESE/TESA: Testicular sperm extraction/aspiration
